# First record of harpacticoid copepods from Lake Tahoe, United States: two new species of *Attheyella* (Harpacticoida, Canthocamptidae)

**DOI:** 10.3897/zookeys.479.8673

**Published:** 2015-01-29

**Authors:** Hyun Woo Bang, Jeffrey G. Baguley, Heejin Moon

**Affiliations:** 1 Department of Biology, University of Nevada Reno, Reno, Nevada 89557, USA

**Keywords:** Benthic Harpacticoida, Canthocamptidae, Lake Tahoe, Nevada, California

## Abstract

Benthic harpacticoids were collected for the first time at Lake Tahoe, California-Nevada, United States. Two species were identified as members of the genus *Attheyella* Brady, 1880. The genus *Attheyella* comprises about 150 species within six subgenera, but only twelve species have previously been reported from North American freshwater habitats. The two new species of *Attheyella* described here have a 3-segmented endopod on P1 and 2-segmented P2–P4 endopods, the distal segment of exopod of P2–P4 has three outer spines, and the P5 has five setae on the exopod and six setae on the baseoendopod. Attheyella (Attheyella) tahoensis **sp. n.** most closely resembles Attheyella (Attheyella) idahoensis (Marsh, 1903) from Idaho, Montana, and Alaska (United States) and Attheyella (Attheyella) namkungi Kim, Soh & Lee, 2005 from Gosu Cave in South Korea. They differ mainly by the number of setae on the distal endopodal segment of P2–P4. In addition, intraspecific variation has been observed on the caudal rami. Attheyella (Neomrazekiella) tessiae **sp. n.** is characterized by the extension of P5 baseoendopod, 2-segmented endopod of female P2–P3, and naked third seta of male P5 exopod. The two new species are likely endemic to Lake Tahoe, an isolated alpine lake within the Great Basin watershed in the western United States.

## Introduction

Lake Tahoe is a large freshwater lake in the Sierra Nevada of the United States. At a surface elevation of 1,897 m, it is located along the border between California and Nevada. Lake Tahoe is the largest alpine lake in North America. Its depth is 501 m, making it the deepest in the United States after Crater Lake (593 m). Lake Tahoe is one of the largest lakes by volume (1.5 × 1011 m^3^) in the United States, only being exceeded by the Great Lakes. While, some basic ecological investigations have occurred in Lake Tahoe (Flint and Goldman 1975, Frantz and Cordone 1996, Vander Zanden et al. 2003), most recent investigations are focused on studies of water quality (Jassby et al. 2003), invasive species (Denton et al. 2012, Wittmann et al. 2012), and loss of native biodiversity (Caires et al. 2013). Meiobenthic (especially harpacticoid copepods) diversity has not been studied in Lake Tahoe until recently, and the ecological role of meiobenthos in Lake Tahoe remains unstudied.

During the past century, fresh water harpacticoid copepods in North America have been reported by several researchers, with checklists provided by: Wilson CB (1932; Woods Hole, Massachusetts), Coker (1934; Illinois, North Carolina and Minnesota), Reid (1996; Washington, D.C.), Hudson et al. (1998; the Great Lakes), Suárez-Morales and Reid (1998; the Yucatan peninsula), Bruno et al. (2005; Florida), and Reid and Hribar (2006; the Florida Keys). Moreover, Wilson and Yeatman (1959) and Reid and Williamson (2009) provided key to species in North American freshwater harpacticoid copepods.

Considering all studies of North American harpacticoid copepods, Canthocamptidae is the most abundant harpacticoid family on the continent (Reid and Williamson 2009) with representatives of the following 12 genera; *Attheyella* Brady, 1880, *Bryocamptus* Chappuis, 1928, *Canthocamptus* Westwood, 1836, *Cletocamptus* Schmankevitsch, 1875, *Elaphoidella* Chappuis, 1928, *Epactophanes* Mrázek, 1893, *Gulcamptus* Miura, 1969, *Heteropsyllus* Scott T., 1894, *Maraenobiotus* Mrázek, 1893, *Mesochra* Boeck, 1865, *Moraria* Scott T. & Scott A., 1893, and *Pesceus* Özdikmen, 2008 (Reid and Williamson 2009).

The genus *Attheyella* Brady, 1880 has been found in a wide range over the world (Dussart and Defaye 1990). Despite its cosmopolitan distribution, only 12 species of *Attheyella* have been reported in North America; Attheyella (Attheyella) alaskaensis Wilson M.S., 1958, Attheyella (Attheyella) idahoensis (Marsh, 1903), Attheyella (Attheyella) obatogamensis (Willey, 1925), Attheyella (Neomrazekiella) americana (Herrick, 1884), Attheyella (Neomrazekiella) dentata (Poggenpol, 1874), Attheyella (Neomrazekiella) dogieli (Rylov, 1923), Attheyella (Neomrazekiella) illinoisensis (Forbes S.A., 1876), Attheyella (Neomrazekiella) nordenskioldii (Lilljeborg, 1902), Attheyella (Neomrazekiella) ussuriensis Rylov, 1933, Attheyella (Ryloviella) carolinensis Chappuis, 1932, Attheyella (Ryloviella) pilosa Chappuis, 1929, and Attheyella (Ryloviella) spinipes Reid, 1987.

As part of ongoing efforts to understand the ecological role of meiobenthos in Lake Tahoe, harpacticoid copepods have been collected and documented here for the first time. Here, two new species of *Attheyella* from the Lakeside Beach littoral zone are described and an updated key to species of *Attheyella* in North America is provided.

## Material and methods

Samples were collected from the Lakeside Beach littoral zone, 5 m water depth, on south shore of Lake Tahoe near the California-Nevada border in July 2013. Sediment samples were collected manually by SCUBA divers. Sediments were sampled with 2.9 cm inner diameter core tubes and were fixed with 70% ethanol and stained with Rose Bengal. Meiofauna was extracted from sediments by Ludox isopycnic centrifugation (Burgess 2001). Due to the coarseness of sand typical of Lake Tahoe, vortexing was replaced with gentle agitation during the Ludox extraction procedure to prevent mechanical damage to animals. Harpacticoids were sorted and enumerated under a Leica S8APO dissecting microscope, and stored in 70% ethanol.

Specimens were dissected in lactic acid and the dissected parts were mounted on slides in lactophenol mounting medium. Preparations were sealed with transparent nail varnish. All drawings have been prepared using a camera lucida on a Leica DM 2500 differential interference contrast microscope. Specimens were deposited at the Smithsonian National Museum of Natural History.

The descriptive terminology is adopted from Huys et al. (1996). Abbreviations used in the text are: A1, antennule; A2, antenna; ae, aesthetasc; exp, exopod; enp, endopod; P1–P6, first to sixth thoracopod; exp (enp)-1 (2, 3) to denote the proximal (middle, distal) segment of a ramus. Scale bars in figures are indicated in μm.

## Results

### Order Harpacticoida Sars, 1903 Family Canthocamptidae Brady, 1880 Genus *Attheyella* Brady, 1880

#### Subgenus Attheyella (Attheyella) Chappuis, 1929

##### Attheyella (Attheyella) tahoensis sp. n.

Taxon classificationAnimaliaHarpacticoidaCanthocamptidae

http://zoobank.org/7FF5E8A1-200A-42FA-8C5C-30F5EB58202B

**Figure 1.**
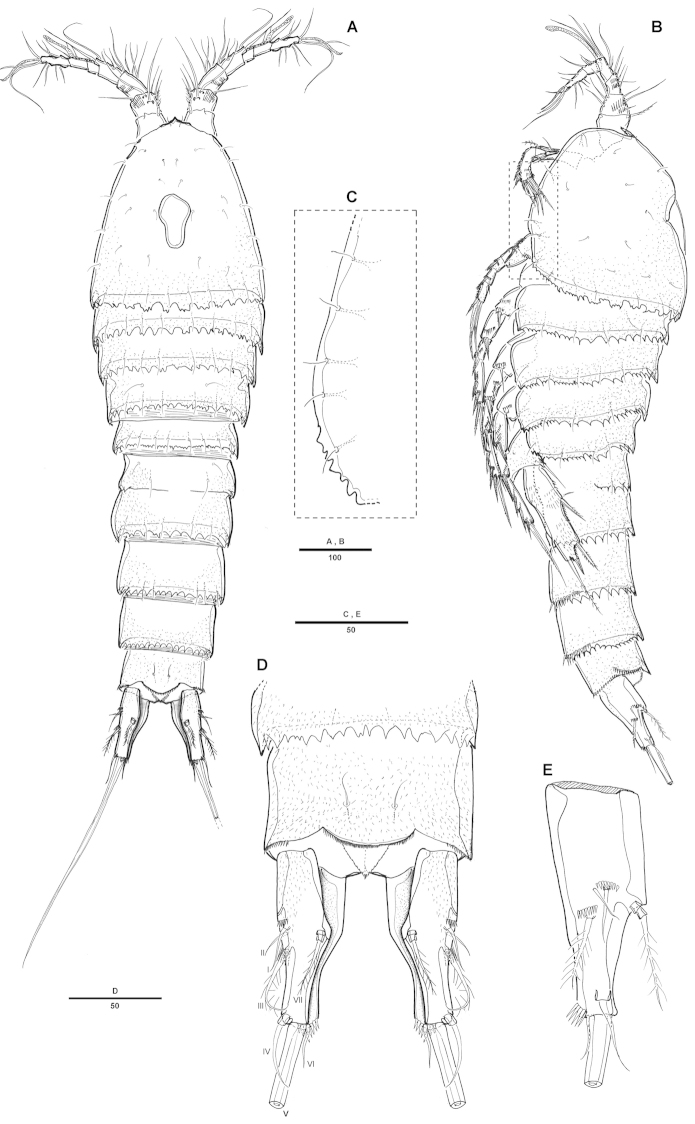
Attheyella (Attheyella) tahoensis sp. n. female: **A** habitus, dorsal **B** habitus, lateral **C** cephalothorax lateral anterior margin **D** anal somite and caudal rami, dorsal **E** caudal ramus, lateral.

**Figure 2.**
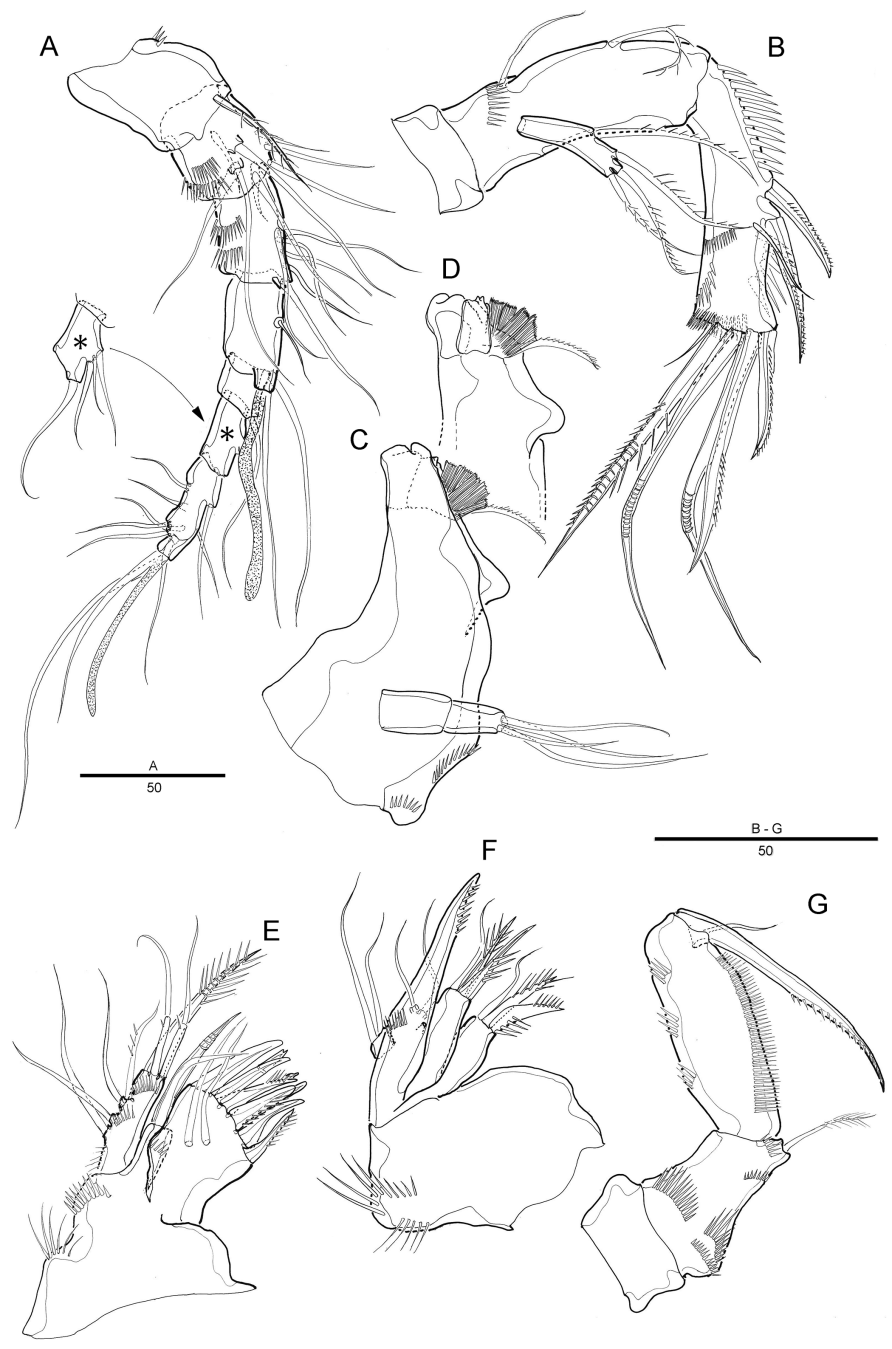
Attheyella (Attheyella) tahoensis sp. n. female: **A** antennule **B** antenna **C** mandible **D** mandible, other view **E** maxillule **F** maxilla **G** maxilliped.

**Figure 3.**
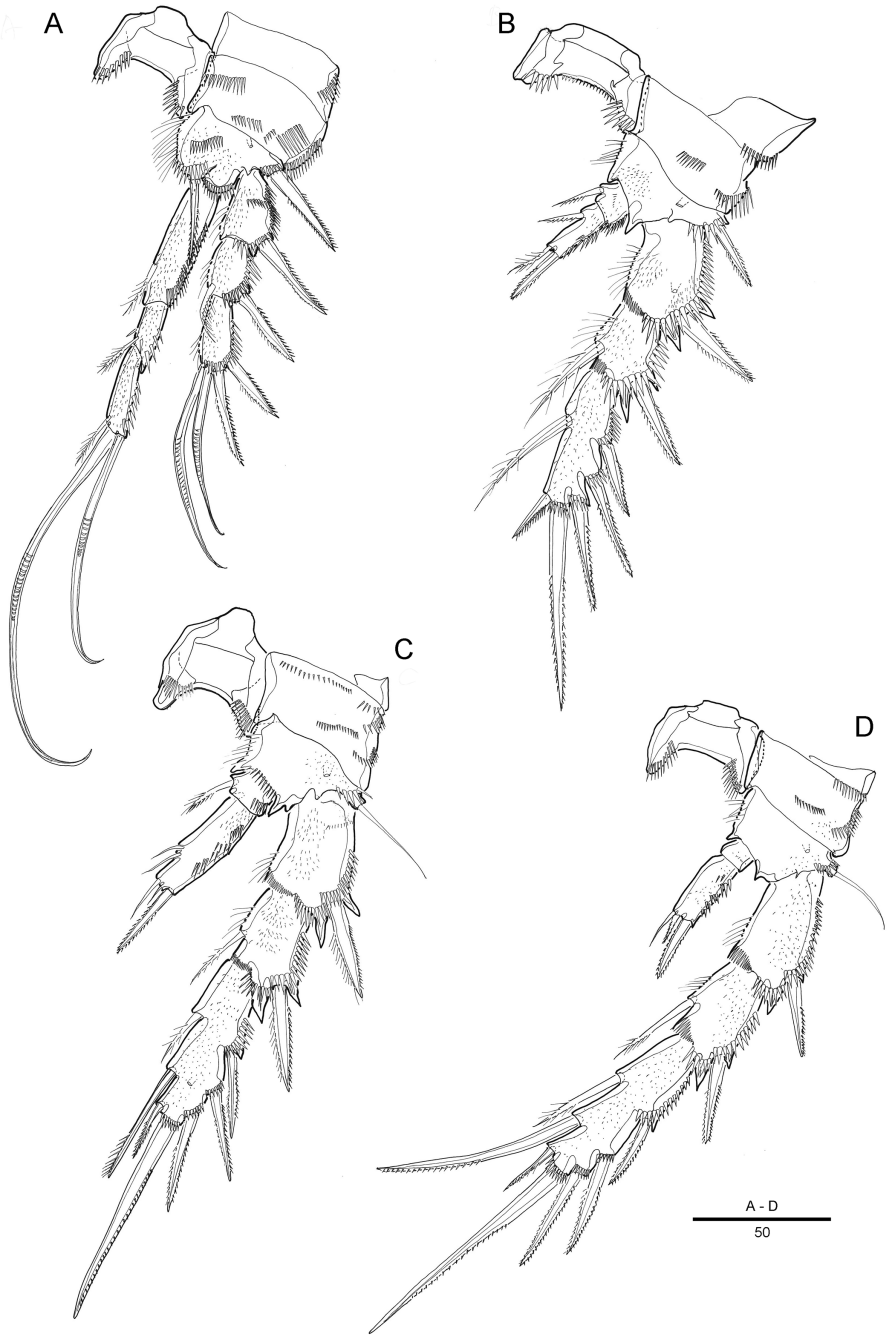
Attheyella (Attheyella) tahoensis sp. n. female: **A** P1, anterior **B** P2, anterior **C** P3, anterior **D** P4, anterior.

**Figure 4.**
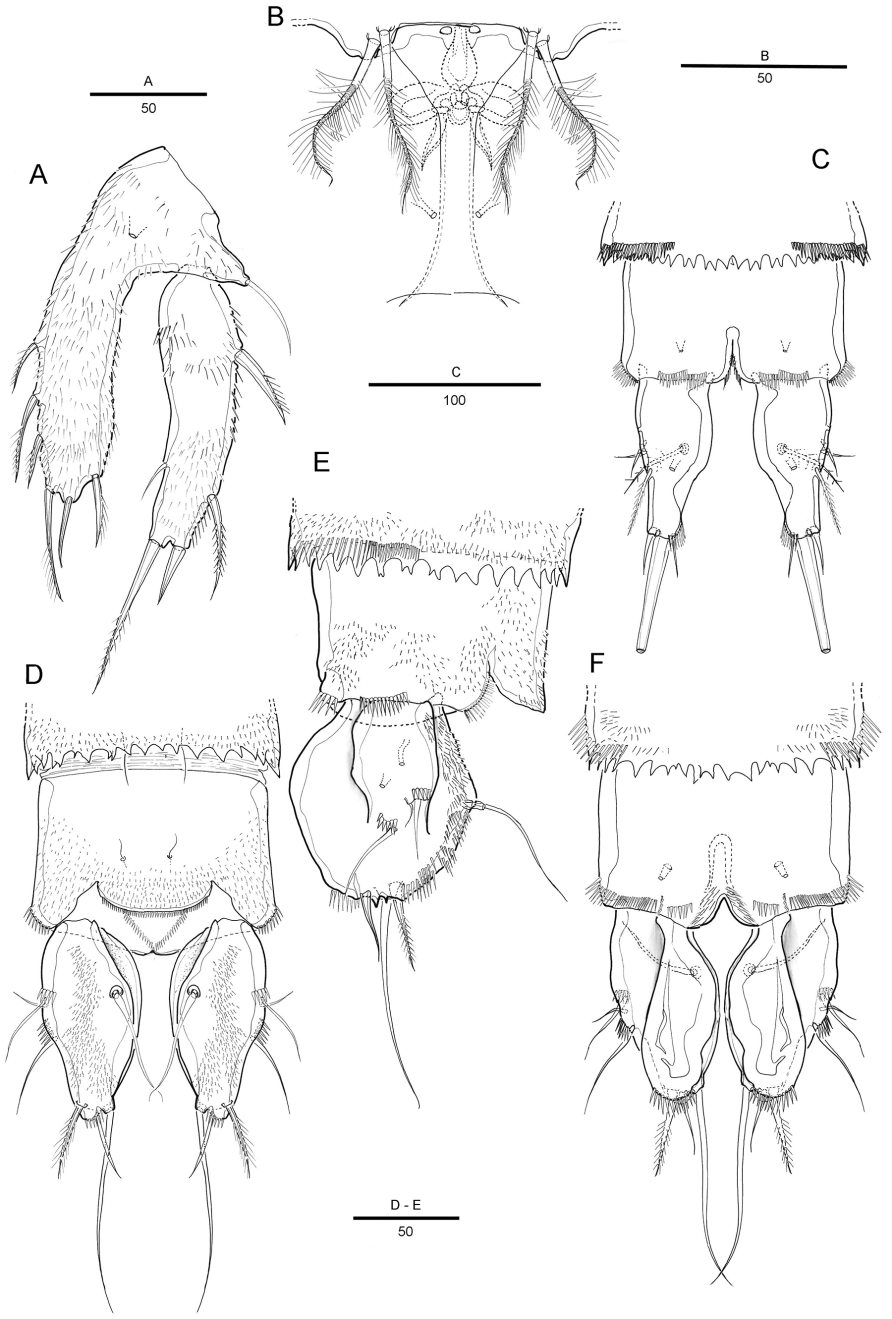
Attheyella (Attheyella) tahoensis sp. n. female: **A** P5, anterior **B** genital field, ventral **C** anal somite and caudal rami, ventral **D** abnormal caudal rami, dorsal **E** abnormal caudal rami, lateral **F** abnormal caudal rami, ventral.

**Figure 5.**
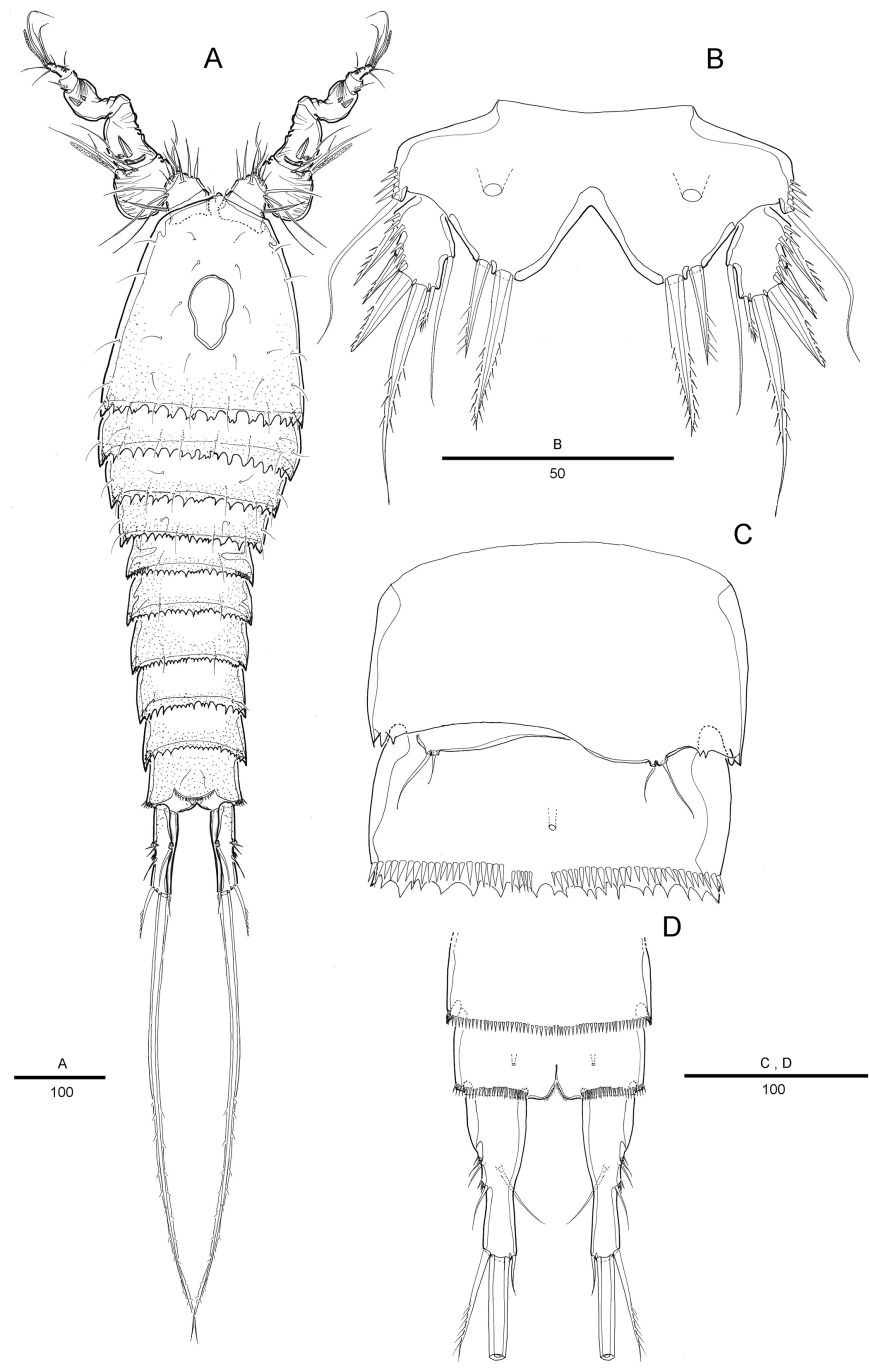
Attheyella (Attheyella) tahoensis sp. n. male: **A** habitus, dorsal **B** P5, anterior **C** genital filed, ventral **D** anal somite and caudal rami, ventral.

**Figure 6.**
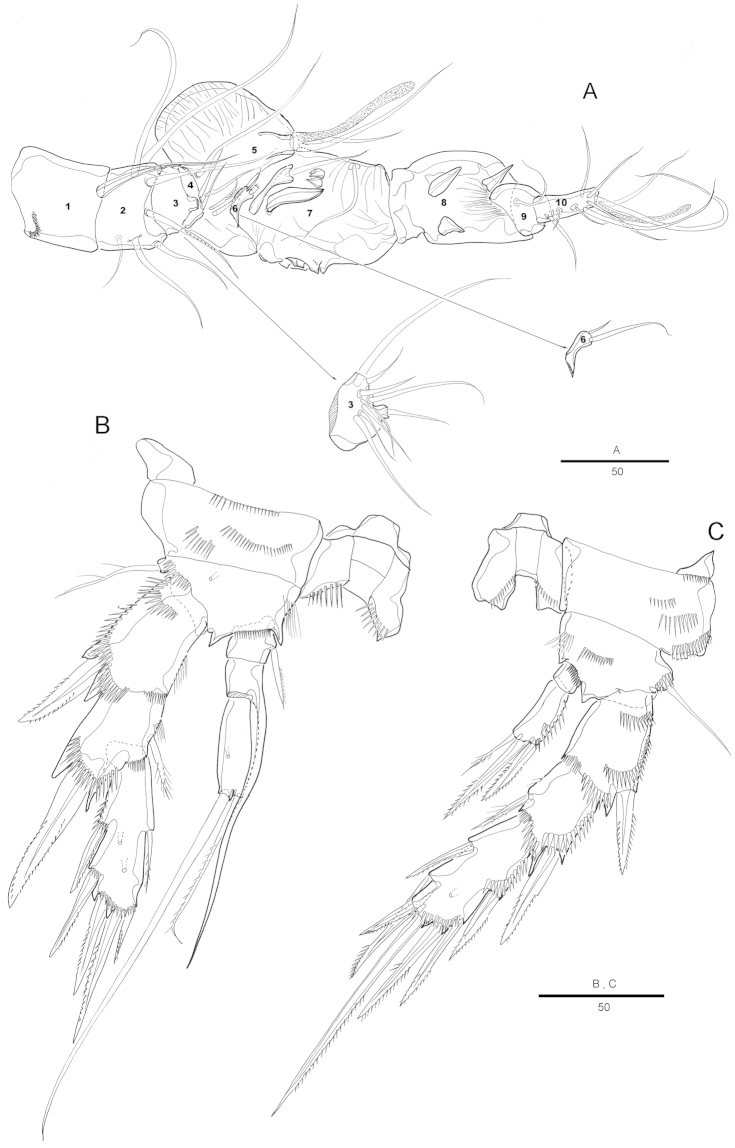
Attheyella (Attheyella) tahoensis sp. n. male: **A** antennule **B** P3, anterior **C** P4, anterior.

Figs 1 , 2 , 3 , 4 , 5 , 6

###### Type locality.

The Lakeside littoral zone; 38°57'42"N, 119°57'14"W, 5 m water depth, of Lake Tahoe in California-Nevada, United States.

###### Material examined.

Holotype: 1♀ (USNM No: 1251801) dissected on 8 slides. Paratype 2♀♀ and 1♂ (USNM No’s listed in order presented in text: 1251802, 1251804, 1251803) each dissected on 6, 9 and 8 slides respectively, and 6♀♀ and 4♂♂ (USNM No’s for specimens in vials (female, male): 1251805, 1251806) in 70% ethanol, vial. All from the type locality, July 2013, *leg.* J.G. Baguley.

###### Description.

Female. Total body length 816 µm (n=6; range: 753-868 µm), measured from anterior margin of rostrum to posterior margin of caudal rami. Largest width measured at posterior margin of cephalic shield: 241 µm. Urosome narrower than prosome (Fig. 1A). Posterior and lateral margins on all somites except anal somite strongly serrated (Fig. 1A–B).

Cephalothorax (Fig. 1A) bell-shaped, with saddle-shaped dorsal integumental window and some scattering sensillae on dorsal surface and along lateral margin (Fig. 1C). Pedigerous somites with sensillae on dorsal surface, serrate posteriorly as cephalothorax; pleural areas well developed. Urosome 5-segmented, comprising P5-bearing somite, genital double-somite and 3 free abdominal somites.

Genital somite and first abdominal somite partly fused forming double-somite, wider than long. Genital field as in Fig. 4B. Genital apertures located anteriorly, closely set together. Copulatory pore located anteriorly between genital apertures. Seminal receptacle well developed on each side. P6 with small protuberance bearing 2 pinnate setae.

Anal somite (Figs 1D, 4C) with pair of sensilla dorsally, with well-developed rounded operculum bearing row of setules. Caudal rami (Fig. 1D–E) bottle-shaped, strongly tapering distally, about 2.5 times as long as wide, each ramus with 7 setae: setae I-II bare, short, of subequal lengths, closely set, seta III bipinnate, seta IV bare, seta V longest, seta VI bare and short, seta VII tri-articulate at base. Inner margin of each ramus with lateral concavity.

Antennule (Fig. 2A) 7-segmented. Segment 1 largest, with 1 spinular row and 1 seta. Segment 2 with 2 spinular rows around posterior and lateral margins. Segment 4 with aesthetasc fused basally to seta and set on pedestal. Armature formula: 1-[1], 2-[9], 3-[6], 4-[2+(1+ae)], 5-[1], 6-[4], 7-[8+acrothek]. Apical acrothek consisting of a small aesthetasc fused basally to 2 bare setae.

Antenna (Fig. 2B) 3-segmented, comprising coxa, allobasis and free 1-segmented endopod. Coxa small. Allobasis elongated; spinules on abexopodal margin; with 2 long abexopodal setae. Exopod 1-segmented; with 1 inner and 3 apical pinnate setae. Endopod elongated, with strong spinules along inner margin; lateral armature consisting of 2 pinnate spines and a minute seta; distal armature consisting of 2 apically curved pinnate spines and 3 geniculate setae, the outer-most bipinnate and basally fused to an additional short seta.

Mandible (Fig. 2C–D) with well-developed gnathobase bearing 2 strong teeth and several smaller, multicuspidate teeth around distal margin and 1 pinnate spine at dorsal corner; spinules near base of palp. Palp 2-segmented, distal segment with 4 bare setae.

Maxillule (Fig. 2E). Praecoxal arthrite well developed, with 9 apical strong and transformed spines, 2 bare setae on anterior surface and, few spinules near outer margin. Coxa with cylindrical endite bearing 1 naked seta, and 1 curved spine. Basis with 2 geniculated setae and 1 bipinnate spine apically; with several spinules around inner distal margin and base of endopod. Endopod and exopod incorporated in basis and presented by 1 pinnate and 1 naked seta, and 2 naked setae, respectively.

Maxilla (Fig. 2F). Syncoxa with 2 endites each carrying apically 2 strong pinnate spines and 1 seta. Allobasis drawn out into strong, slightly curved, distally pinnate claw, accessory armature consisting of 2 bare setae with 1 tube pore. Endopod small, with 2 naked setae.

Maxilliped (Fig. 2G). Syncoxa with 1 plumose seta on inner distal corner. Basis with 1 row of spinules along palmar region. Endopodal segment produced into strong and distally pinnate curved claw; accessory armature consisting of 1 small seta at base.

Swimming legs 1-4 with wide intercoxal sclerite, biramous, endopods 2-segmented except for P1, exopods 3-segmented. Coxa and basis with row of spinules along outer margins as illustrated.

P1 (Fig. 3A). Preacoxa large, with longitudinal spinular row on anterior surface. Coxa large, with four spinular rows on anterior surface, and row of spinules along outer margin. Basis with strong bipinnate outer spine on outer margin and bipinnate spine on inner distal surface, with several spinules and setules as figured. Endopod 3-segmented; enp-1 about 2.1 times as long as enp-2; enp-1 with one small inner bipinnate seta on distal fourth; enp-2 with 1 bipinnate inner seta; enp-3 with 1 small pinnate seta and 2 long geniculate setae distally. Exopod 3-segmented, reaching middle of enp-2, exp-2 with 1 inner pinnate seta; exp-3 with 2 geniculate distal setae and 2 strong spinulose outer spines.

P2–P4. Coxa and basis with spinular rows along outer margin and anterior surface. Basis with pinnate spine (P2) or bare seta (P3–P4), each seta arising from a setophore.

P2 (Fig. 3B) with large coxa, ornamented with row of spinules on anterior surface, and with row of long spinules along outer margin; P2 enp-2 more than twice as long as enp-1; with 1 short, pinnate inner seta; enp-2 with 1 inner pinnate short seta, and 1 short apical seta and 1 bipinnate apical spine; exopod 3-segmented; each segment with row of spinules along outer margins; third segment about 1.8 times as long as second segment with 3 strong bipinnate outer spines, 2 apical pinnate spines, and 1 inner bipinnate seta.

P3 (Fig. 3C) with small praecoxa. Coxa nearly 1.5 times as wide as long, with 3 spinular rows on anterior surface and 2 rows of spinules along outer margin. Enp-2 about 3 times longer than enp-1; enp-2 with 2 small bare inner setae and 2 short distal naked setae and one pinnate spine.

P4 (Fig. 3D) with small and triangular praecoxa, with row of spinules on anterior surface along distal margin. Coxa with 1 row of small spinules on anterior surface and 1 spinular row along outer margin. Enp-2 2.5 times as long as enp-1; enp-2 with 1 naked seta, 1 pinnate seta and 1 spine. Spine and setal formulae as follows:

**Spine and setal formulae:** 

	Exopod	Endopod
P1	0.1.022	1.1.120
P2	0.1.123	1.120
P3	0.1.223	1.230
P4	0.1.223	0.030

P5 (Fig. 4A) with separate exopod and baseoendopod, each covered with spinules as illustrated. Baseoendopod longer than wide, forming short outer setophore bearing the basal seta. Endopodal lobe long and almost reaching distal margin of exopod, with 3 pinnate inner setae, 2 distal setae, and 1 pinnate outer seta. All setae pinnate and short. Secretory pore on anterior surface. Exopod elongated, 3.4 times as long as wide, with 1 short inner, 2 distal and 2 outer setae.

P6 (Fig. 4B) each with small protuberance bearing 2 plumose setae.

###### Description.

Male. Body slightly smaller and more slender than female, habitus as in Fig. 5A. Body length 784 µm (n=5; range: 765-821 µm), measured from anterior margin of rostrum to posterior margin of caudal rami. Largest width measured at P2-bearing somite: 214 µm. Sexual dimorphism in antennule, P3-P4 endopod, P5 and P6.

Prosome (Fig. 5A) posterior margin of cephalothorax and pedigerous somites with serrated process, with integumental sensilla.

Urosome (Fig. 5A, C) 6-segmented, comprised of P5-bearing somite, genital somite, and 4 free abdominal somites. Urosomite with serrated posterior margin dorsally and ventrally.

Caudal rami (Fig. 5D) slightly more elongated than female, about 3.3 times as long as wide, seta III bare, seta IV pinnate. Inner margin of each ramus with lateral concavity.

Antennule (Fig. 6A) 10-segmented; subchirocer with geniculation between segments 5 and 6, and between segment 7 and 8. Segment 1 with a row of spinules along anterior margin. Segment 4 and 6 represented by a small sclerite. Segment 5 swollen with large bump along posterior margin. Segment 7 and 8 with 3 spinular processes from modified setae on each segment. Armature formula: 1-[1], 2-[7], 3-[9], 4-[2], 5-[5 + (1 + ae)], 6-[2], 7-[2 + 3 modified] 8-[3 modified], 9-[1], 10-[7 + acrothek]. Apical acrothek consisting of a small aesthetasc fused basally to 2 bare setae.

P3 (Fig. 6B). Exopod as in female, except for outer spine on first and second exopodal segment of P3 proportionately stronger. Endopod modified, 3-segmented; enp-1 shortest with inner pinnate seta; enp-2 with well-developed inner apophysis; enp-3 with 1 pinnate apical seta and 1 long bare seta.

P4 (Fig. 6C). Setae and spines on exopod modified, outer spine on first and second exopodal segment proportionally stronger than female. Endopod 2 with 1 pinnate seta and 2 pinnate spines and longer than those in female.

Fifth pair of legs (P5) (Fig. 5B) smaller and much shorter than female and fused medially, with no spinules. Baseoendopod with outer setophore bearing the basal seta. Endopodal lobe with 2 distal pinnate setae with large pore on anterior surface. Exopod shorter than in female, as long as wide, with 1 inner bare seta, 2 distal pinnate setae and 1 pinnate spine, and 2 outer pinnate spines.

P6 (Fig. 5C) asymmetrical, bearing 2 naked seta on a cylindrical process. On left side a lobe with two setae, on the right side a small plate with two setae.

###### Variability.

Intraspecific variability was observed in the shape of caudal rami of female (about 20%; 6/30 observed individuals). Caudal rami (Fig. 4D–F) lamelliform and elongate, about twice as long as wide, laterally compressed, inner margin of each ramus with lateral concavity. Each ramus with 7 setae: seta III bare, seta IV bipinnate, seta V extremely reduced, seta VI bare and longer than normal.

###### Etymology.

The species name refers to the type locality, Lake Tahoe. This is one of the largest alpine lakes in the world is known for its pristine waters and aesthetic beauty.

#### Subgenus Attheyella (Neomrazekiella) Ozdikmen & Pesce, 2006

##### Attheyella (Neomrazekiella) tessiae sp. n.

Taxon classificationAnimaliaHarpacticoidaCanthocamptidae

http://zoobank.org/752396F3-38A4-4B57-B2C9-294B1FC888D8

Figs 7 , 8 , 9 , 10

###### Type locality.

The Lakeside littoral zone; 38°57'42"N, 119°57'14"W, 5 m water depth, of Lake Tahoe in California-Nevada, United States.

###### Material examined.

Holotype: 1♀ (USNM No: 1251796) dissected on 9 slides, from the type locality. Paratype 1♀ and 1♂ (USNM No’s listed in order presented in text: 1251797, 1251798) each dissected on 3 and 9 slides respectively, and 10♀♀ and 6♂♂ (USNM No’s for specimens in vials (female, male): 1251799, 1251800) in 70% ethanol, vial. Additional samples were deposited in the first author’s collection. All from the type locality, July 2013, *leg.* J. G. Baguley.

###### Description.

**Figure 7.**
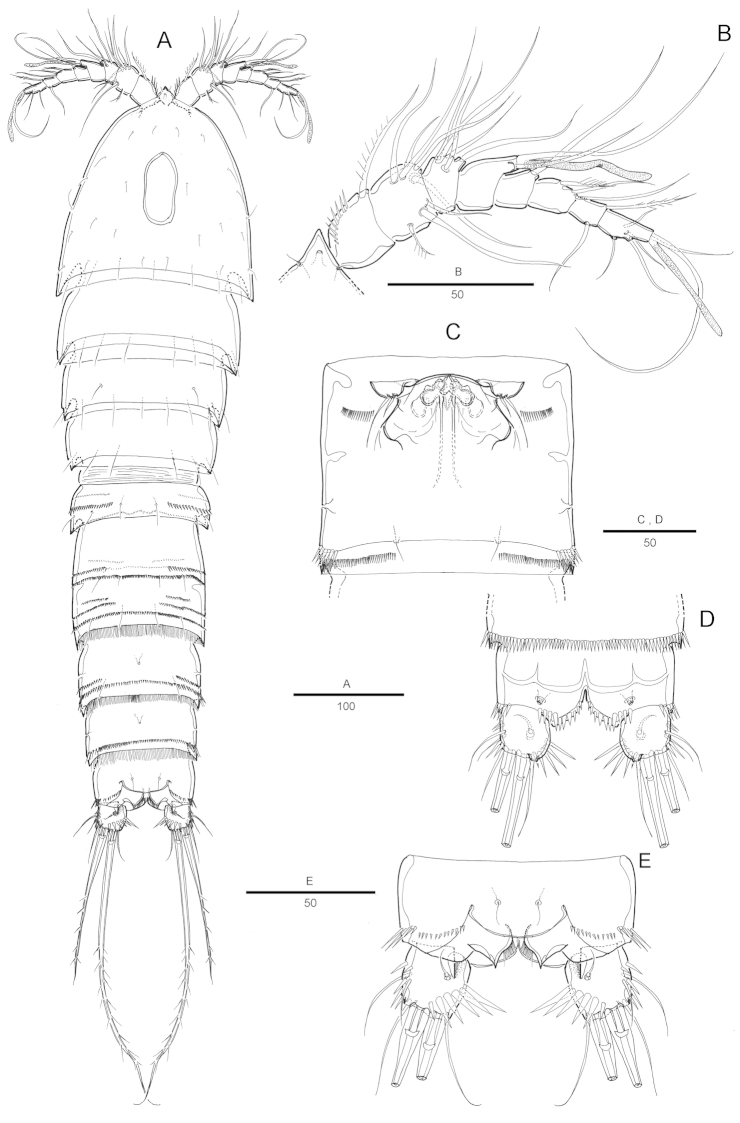
Attheyella (Neomrazekiella) tessiae sp. n. female: **A** habitus, dorsal **B** antennule **C** genital field **D** anal somite and caudal rami, ventral; **E** anal somite and caudal rami, dorsal.

Female. Total body length 660 µm (n=5; range: 635–694 µm), measured from anterior margin of rostrum to posterior margin of caudal rami. Largest width measured at posterior margin of cephalic shield: 176 µm. Urosome narrower than prosome (Fig. 7A).

Cephalothorax (Fig. 7A) bell-shaped, with oval dorsal integumental window and some scattering sensillae on dorsal surface and along lateral margin. Rostrum (Fig. 7B) small and triangular, completely fused to cephalothorax and with pair of sensilla and pore near anterior margin.

Prosome with smooth posterior margins, pleural areas well developed. Body slightly constricted between each somite. All pedigerous somites with plain hyaline frill on posterior margin. Urosome 5-segmented, comprising P5-bearing somite, genital double-somite and 3 free abdominal somites.

Genital double-somite as wide as long. Original segmentation marked by discontinuous internal chitinous rib laterally, completely fused ventrally. A row of spinules present on lateral side of genital field. Genital field located far anteriorly (Fig. 7C). Genital apertures paired, closely set together. Copulatory pore located anteriorly between genital apertures. Seminal receptacle well developed on each side. P6 with small protuberance bearing 2 bare unequal setae.

Anal somite (Fig. 7D–E) with well-developed rounded operculum. Surface ornamentation consisting of a pair of sensilla dorsally and a pair of pores ventrally, posterior margin with spinules ventrally and dorsally, with triangular process dorsally, anal opening with a fringe of fine setules. Caudal rami short, as long as wide, each ramus with 7 setae: setae I-II small, closely set, seta III bare, seta IV pinnate, seta V bipinnate and longest, seta VI bare, seta VII tri-articulate at base.

Antennule (Fig. 7B) 8-segmented. Segment 1 largest, with 1 spinular row around posterior margin and 1 pinnate seta. Segment 4 with aesthetasc fused basally to seta and arising from a pedestal. Armature formula: 1-[1], 2-[8], 3-[5], 4-[1+(1+ae)], 5-[1], 6-[3], 7-[2], 8-[4+acrothek]. Apical acrothek consisting of a small aesthetasc fused basally to 2 bare setae.

Antenna, mandible, maxillule, maxilla, and maxilliped as in Attheyella (Attheyella) tahoensis sp. n.

**Figure 8.**
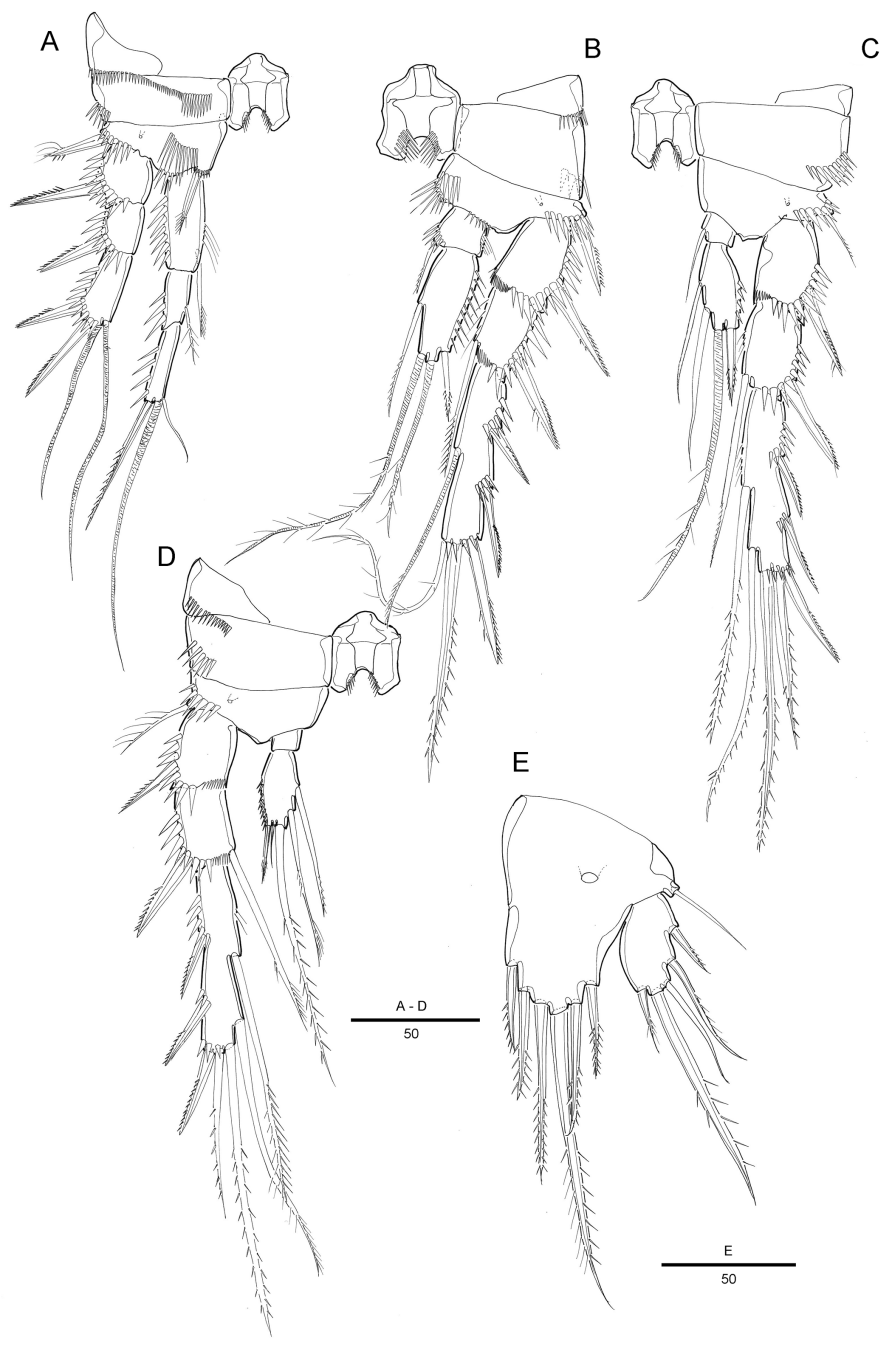
Attheyella (Neomrazekiella) tessiae sp. n. female: **A** P1, anterior **B** P2, anterior **C** P3, anterior **D** P4, anterior **E** P5, anterior.

P1 (Fig. 8A) with well-developed coxa with spinular row on anterior surface. Basis with setules along outer margin and anterior surface, with pinnate outer and inner spines. Endopod 3-segmented, 1.3 times as long as exopod; enp-1 longer than enp-2, with one inner pinnate seta; enp-2 with 1 bipinnate inner seta; enp-3 with a small inner seta, and 1 long geniculate seta and 1 pinnate seta distally. Exopod 3-segmented, reaching end of enp-2, exopodal segments with strong spinules along outer margin and outer distal corner; exp-3 with 2 geniculate distal setae and 2 strong spinulose outer spines.

P2 (Fig. 8B) with small triangular praecoxa, with row of spinules on anterior surface along distal margin. Coxa nearly 1.5 times as wide as long, ornamented with row of long spinules along outer margin; Basis with outer pinnate spine; P2 enp-2 more than twice as long as enp-1; with 1 short, pinnate inner seta; enp-2 with 1 inner pinnate seta, and 2 distal bipinnate setae and 1 bipinnate outer seta; exopod 3-segmented; each segment with row of spinules along outer margins; third segment about 2.3 times as long as second segment with 3 bipinnate outer spines, 1 apical pinnate spines and 1 plumose seta, and 1 inner long bipinnate seta.

P3 (Fig. 8C) with small praecoxa. Coxa nearly 1.5 times as wide as long, with row of spinules along outer margin. Basis with outer pinnate seta and spinular row along outer margin. Endopod-1 with 1 bare inner seta; enp-2 with 2 inner naked setae, and 1 pinnate seta and short distal spine; exp-3 about twice as long as second segment with 3 bipinnate outer spines, 2 apical pinnate spines and 2 long bipinnate inner setae.

P4 (Fig. 8D) with small and triangular praecoxa. Coxa with spinular row along outer margin. Enp-2 with 2 inner pinnate setae, and 3 pinnate setae, the innermost longest; exp-3 with 3 bipinnate outer spines, 2 apical pinnate setae and 2 long bipinnate inner setae. Spine and setal formulae as follows:

**Spine and setal formulae:** 

	Exopod	Endopod
P1	0.0.022	1.1.120
P2	0.1.123	1.121
P3	0.1.223	1.220
P4	0.1.223	0.230

P5 (Fig. 8E). Baseoendopod forming short, outer setophore bearing the basal seta. Endopodal lobe trapezoidal, with 2 pinnate inner setae, 2 distal setae, and 2 pinnate outer seta; all setae of different length and apical outermost is the longest. A secretory pore on anterior surface. Exopod twice as long as wide, with one short inner, 2 distal (innermost longest) and 2 outer setae, all pinnate.

###### Description.

**Figure 9.**
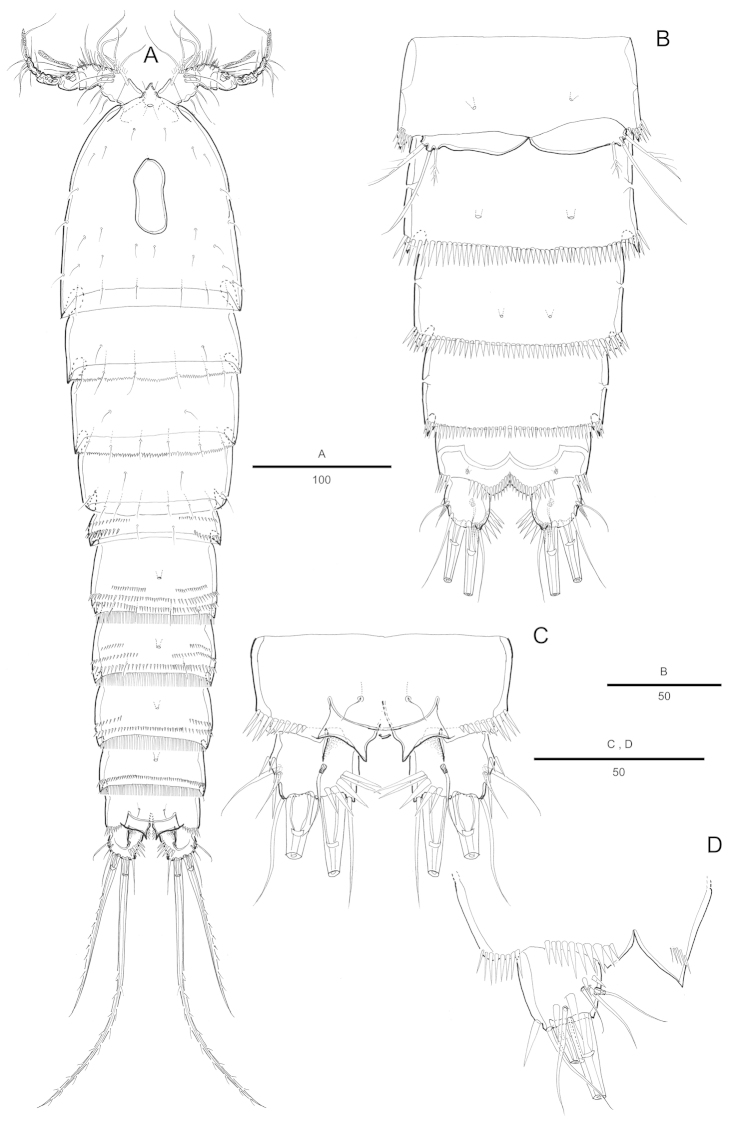
Attheyella (Neomrazekiella) tessiae sp. n. male: **A** habitus, dorsal **B** Urosome (excluding P5-bearing somite), ventral **C** anal somite and caudal rami, dorsal; **E** anal somite and caudal ramus, lateral.

Male (Fig. 9A). Body smaller and more slender than female. Body length 564 µm (n=6; range: 509–613 µm), measured from anterior margin of rostrum to posterior margin of caudal rami. Largest width measured at P2-bearing somite: 131 µm. Sexual dimorphism in antennule, P3-P4 endopod, P5 and P6.

Cephalothorax (Fig. 9A) with smooth posterior margin, with integumental sensilla. Urosome (Fig. 9B–D) 6-segmented, comprised of P5-bearing somite, genital somite, and 4 free abdominal somites. Urosomites with spinules along posterior margin dorsally and ventrally. Anal somite with inner process on lateral margin.

**Figure 10.**
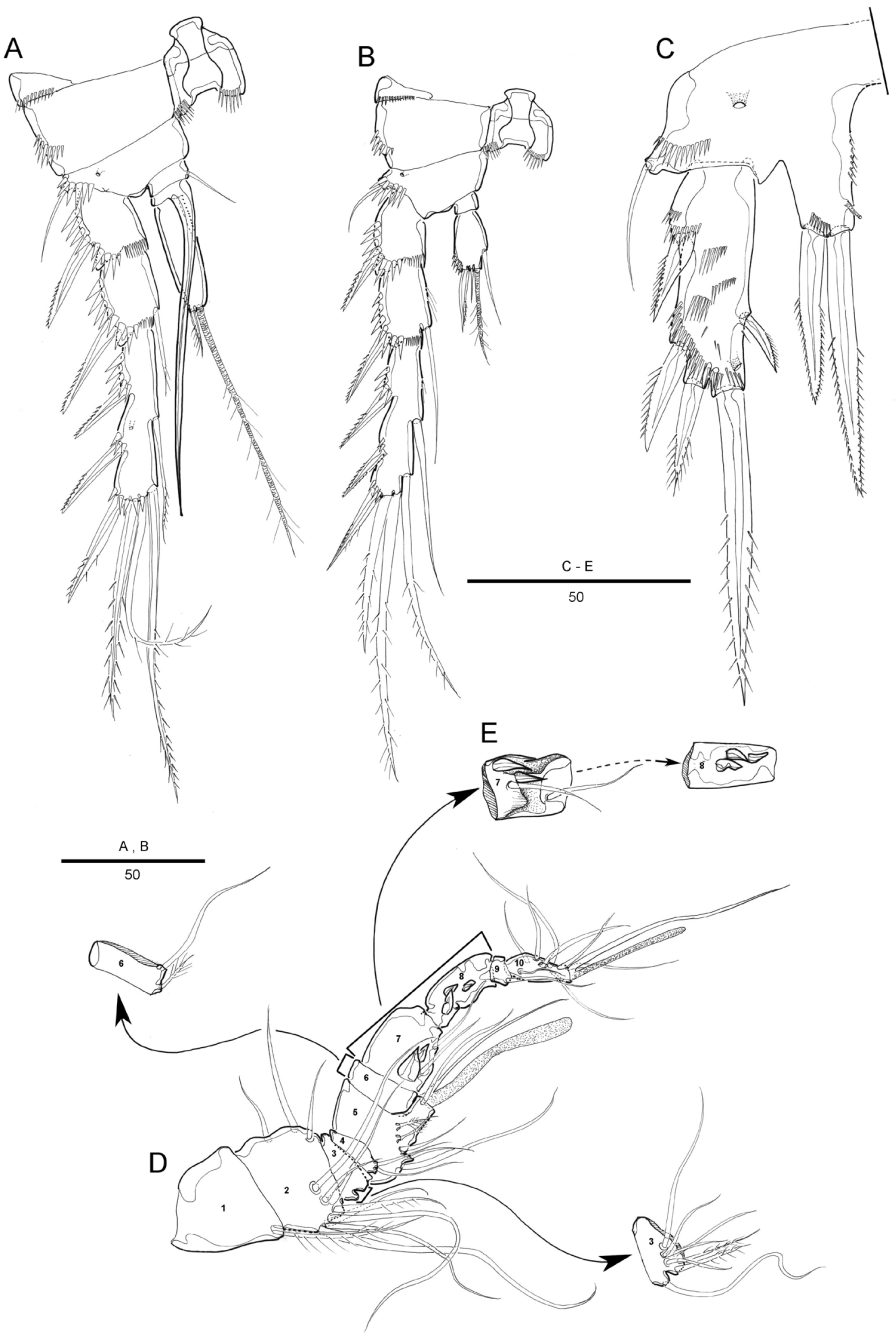
Attheyella (Neomrazekiella) tessiae sp. n. male: **A** P3, anterior **B** P4, anterior **C** P5, anterior **D** antennule **E** seventh and eighth segments of antennule, other view.

Antennule (Fig. 10D–E) 10-segmented; subchirocer with geniculation between segments 7 and 8. Segment 2 largest. Segment 5 not swollen. Aesthetasc on segments 5 and 10. Some elements on segments 7 and 8. Armature formula: 1-[1], 2-[10], 3-[8], 4-[2], 5-[6 + (1 + ae)], 6-[2], 7-[2 + 2 modified] 8-[3 modified], 9-[1], 10-[7 + acrothek]. Apical acrothek consisting of a small aesthetasc fused basally to 2 bare setae.

P3 (Fig. 10A). Setae on exopod modified. Endopod modified, 3-segmented; enp-1 with inner seta; enp-2 with well-developed inner apophysis; enp-3 with 2 apical setae.

P4 (Fig. 10B). Exp-3 setae modified. Enp-2 with 1 inner and 3 distal setae.

Fifth pair of legs (P5) (Fig. 10C) fused medially. Baseoendopod with outer setophore bearing the basal seta. Endopodal lobe with 2 distal pinnate setae, the outmost longest, large pore on anterior surface. Exopod about 2.6 times as long as wide, with 1 outer and 1 outer distal pinnate setae of similar length, 2 pinnate distal setae, the innermost longest, and a small outer pinnate seta.

P6 (Fig. 9B) asymmetrical, bearing 1 pinnate outer, 1 long naked and 1 short bipinnate inner setae on a cylindrical process, apically.

###### Etymology.

The species name refers to Tahoe Tessie, a cryptozoological creature which supposedly resides in Lake Tahoe. While some claim to have seen the mythical Tahoe Tessie, none until now have seen these non-mythical microscopic creatures of the sand.

## Discussion

The family Canthocamptidae is the largest family of Harpacticoida found in freshwater habitats, and can be found in rivers, streams, ponds, lakes, and wetland, and even in hot springs, glacial melt water and damp moss (Boxshall and Halsey 2004). Canthocamptidae is in need of revision because several genera have high species diversity and many species exhibit wide variation, the widest variability recorded for freshwater Harpacticoida. Additionally, many species are incompletely described, often with major features such as A2 exopod setation and the setal formula for P2–P4 being unknown (Wells 2007).

The genus *Attheyella* Brady, 1880 is a genus of Canthocamptidae, and is cosmopolitan in distribution at the generic level (Boxshall and Halsey 2004). *Attheyella* is highly diverse, with more than 150 species, and is divided into six subgenera: *Attheyella, Canthosella, Chappuisiella, Delachauxiella, Neomrazekiella*, and *Ryloviella*. The genus *Attheyella* is also in need of revision because the species boundaries are not clear due to high variability in number of characters (Reid and Williamson 2009).

Both new species are placed in the genus *Attheyella* because of the following diagnostic features: small rostrum, P1 with 3-segmented rami, 2-segmented endopod of P2–P3 in female, and 3-segmented P3 in males.

In 1876, Forbes S.A. reported the freshwater harpacticoid copepod *Canthocamptus illinoisensis* from Normal, Illinois, which is represented as Attheyella (Neomrazekiella) illinoisensis. Since then, several new species were added, and the genus *Attheyella* in North America currently includes 3 subgenera (*Attheyella, Neomrazekiella, Ryloviella*) and 12 species. Wilson and Yeatman (1959) and Reid and Williamson (2009) provided a key to the species of the genus *Attheyella* from North America. The accepted species are: Attheyella (Attheyella) alaskaensis, Attheyella (Attheyella) idahoensis, Attheyella (Attheyella) obatogamensis, Attheyella (Neomrazekiella) americana, Attheyella (Neomrazekiella) dentata, Attheyella (Neomrazekiella) dogieli, Attheyella (Neomrazekiella) illinoisensis, Attheyella (Neomrazekiella) nordenskioldii, Attheyella (Neomrazekiella) ussuriensis, *A. (Ryloviella) carolinensis, A.* (*Ryloviella*) *pilosa*, and Attheyella (Ryloviella) spinipes.

Attheyella (Attheyella) tahoensis sp. n. clearly belongs to the subgenus *Attheyella* given the elongate exopod and baseoendopod of P5, about equally wide, baseoendopod reaching near the end of exopod in female, and male P3 exopod-2 with enlarged outer spine reaching to end of exopod. Attheyella (Attheyella) tahoensis sp. n. is closely related to the North American Attheyella (Attheyella) idahoensis (Marsh, 1903) and Attheyella (Attheyella) namkungi Kim, Soh and Lee, 2005 from South Korea, with seta formula of the female P4 exopod, and concave shape of caudal rami without a process. However, Attheyella (Attheyella) tahoensis sp. n. can readily be distinguished from its congeners by the number of setae on P2–P4. Attheyella (Attheyella) idahoensis has 4, 5 and 3 setae on distal segment of P2–P4 endopod, whereas Attheyella (Attheyella) tahoensis sp. n. has 3, 4 and 5 setae, respectively. Additionally, Attheyella (Attheyella) namkungi has 3, 4 and 2 setae on the distal endopod of P2–P4.

Morphological variation and abnormality are common in harpacticoid copepods. In the present study, intraspecific variation of Attheyella (Attheyella) tahoensis sp. n. has been recorded, with some specimens having variations on the shape and armature of the caudal rami (about 20% of total observed specimens). In the most commonly observed condition, caudal rami are about 2.5 times as long as wide, bottle-shaped, strongly tapering distally. In the less common morphological variant, caudal rami are about twice as long as wide, laterally compressed, with the inner margin of each ramus having lateral concavity.

In numerous instances, the morphological variation or deformity occurred due to environmental factors such as water pollution. However, some studies suggest that variation in caudal rami may be caused by interspecific competition. For example, Ishida (1994) reported high proportions of caudal rami variation in Attheyella (Attheyella) nakaii (Chappuis, 1927), and suggested that it played a role in sexual segregation due to sympatric distribution with Attheyella (Attheyella) yesoensis Ishida, 1993. Certainly, morphological variation in other harpacticoid species has led to discoveries of concomitant genetic differences and presumed cryptic speciation (Garlitska et al. 2012).

Attheyella (Neomrazekiella) tessiae sp. n. is placed in the subgenus *Neomrazekiella* on account of the following combination of characters: prosome with smooth posterior margins, female P5 endopodal lobe triangular, basal expansion wider than exopod, with six setae, male P5 not produced into narrow prolongation, P3 spine of exopod 2 not greatly enlarged in male. Attheyella (Neomrazekiella) tessiae sp. n. can be clearly distinguished from other members of the subgenus *Neomrazekiella* by the 2-segmented endopods of female P2–P3, P5 baseoendopod produced to middle of exopod segment in female, and naked third seta of the male P5 exopod.

This investigation marks the first record of meiobenthos, and more specifically, of harpacticoid copepods, in Lake Tahoe. The newly described Attheyella (Attheyella) tahoensis sp. n. and Attheyella (Neomrazekiella) tessiae sp. n. are likely endemic to Lake Tahoe. A total of 10 endemic macrobenthos have previously been identified in Lake Tahoe (summarized by Caires et al. 2013), so it is reasonable to hypothesize that several species of meiobenthos also evolved in this ecosystem. Expanded sampling in the western United States, and beyond, will be necessary to validate the endemism of these species.

Together with newly described Attheyella (Attheyella) tahoensis sp. n. and Attheyella (Neomrazekiella) tessiae sp. n., the three subgenera and fourteen species currently recognized as valid in the genus *Attheyella* from North America can be identified with the specific key given below. It is amended from Wilson and Yeatman (1959) and Reid and Williamson (2009).

### Key to the species of the genus *Attheyella* from North America

**Table:** 

1	Female P5 both exopod and baseoendopod elongate, of nearly same width, baseoendopod reaching nearly to end of exopod	**subgenus *Attheyella*... 2**
–	Female P5 baseoendopod much wider than exopod	**5**
2	Caudal ramus inner margin smoothly tapering or concave, without a process	**3**
–	Female caudal ramus with prominent, acute, haired inner process; male caudal ramus with smaller, smooth inner process	**Attheyella (Attheyella) obatogamensis (Willey, 1925)**
3	Caudal ramus narrowed distally, the apex truncate	**4**
–	Caudal ramus hardly at all narrowed distally, the apex rounded	**Attheyella (Attheyella) alaskaensis M.S. Wilson, 1958**
4	Female antennule 8-segmented, P2–P4 endopod-2 with 4, 5, and 3 setae, respectively	**Attheyella (Attheyella) idahoensis (Marsh, 1903)**
–	Female antennule 7-segmented, P2–P4 endopod-2 with 3, 4, and 5 setae, respectively	**Attheyella (Attheyella) tahoensis sp. n.**
5	Female P5 baseoendopod with 3 to 5 setae; caudal rami of both sexes similar, and body segments coarsely serrate	**subgenus *Ryloviella*... 6**
–	Female P5 baseoendopod with 6 setae; caudal rami of both sexes different, and body segments weakly serrate or smooth	**subgenus *Neomrazekiella*... 8**
6	All or most setae on P1 - P5 slender	**7**
–	Setae on P1 - P5 short, stout, spiniform	**Attheyella (Ryloviella) spinipes Reid, 1987**
7	P5 exopod about 2 times as long as wide; female P5 baseoendopod with 3 or 4 setae; caudal ramus with 2 or more longitudinal rows of spinules	**Attheyella (Ryloviella) carolinensis Chappuis, 1932**
–	P5 exopod about 1.5 times as long as wide; female P5 baseoendopod with 5 (rarely 4) setae; caudal ramus with 2 or 3 oblique inner rows of hairs	**Attheyella (Ryloviella) pilosa Chappuis, 1929**
8	Female P2–P3 endopods usually 3-segmented; female P5 endopodal lobe produced to middle of exopod segment or beyond; male P5 exopod seta 3 naked, more slender than other setae	**9**
–	Female P2–P3 endopods usually 2-segmented; female P5 endopodal lobe hardly at all produced; male P5 exopod seta 3 usually similar to other setae	**10**
–	Female P2–P3 endopods 2-segmented; female P5 endopodal lobe reaching distal margin of exopod; male P5 exopod seta 3 naked	**Attheyella (Neomrazekiella) tessiae sp. n.**
9	Female caudal ramus, distal half of outer margin strongly constricted, and outer apical seta outbent at base; male P4 exp3 outer distal and apical spines strongly curved	**Attheyella (Neomrazekiella) nordenskioldii (Lilljeborg, 1902)**
–	Female caudal ramus, outer margin evenly rounded, and base of outer apical seta straight; male P4 exp3 outer distal and apical spines straight	**Attheyella (Neomrazekiella) illinoisensis (S. A. Forbes, 1876)**
10	Caudal ramus, lateral setae inserted next to each other	**11**
–	Caudal ramus, insertions of lateral setae well separated	**12**
11	Female P5 baseoendopod with 6 normal setae; caudal ramus, outer distal corner with rounded sclerotized flange overlying bases of apical setae	**Attheyella (Neomrazekiella) dogieli (Rylov, 1923)**
–	P5 baseoendopod with 6 slender spiniform setae, all of them completely fused with baseoendopod; caudal ramus, outer distal corner with only a few spinules	**Attheyella (Neomrazekiella) ussuriensis Rylov, 1933**
12	Caudal ramus about as long as anal somite, smoothly tapering, dorsal surface with prominent subquadrate or crescentic sclerotization distal to dorsal seta	**Attheyella (Neomrazekiella) dentata (Poggenpol, 1874)**
–	Caudal ramus about 1/2 length of anal somite, outer distal margin constricted, dorsal surface with no special structure	**Attheyella (Neomrazekiella) americana (Herrick, 1884)**

## Supplementary Material

XML Treatment for Attheyella (Attheyella) tahoensis

XML Treatment for Attheyella (Neomrazekiella) tessiae
